# Biomechanical energy harvesting from human motion: theory, state of the art, design guidelines, and future directions

**DOI:** 10.1186/1743-0003-8-22

**Published:** 2011-04-26

**Authors:** Raziel Riemer, Amir Shapiro

**Affiliations:** 1 Department of Industrial Engineering and Management, Ben-Gurion University of the Negev, Beer Sheva, Israel; 2 Department of Mechanical Engineering, Ben-Gurion University of the Negev, Beer Sheva, Israel

## Abstract

**Background:**

Biomechanical energy harvesting from human motion presents a promising clean alternative to electrical power supplied by batteries for portable electronic devices and for computerized and motorized prosthetics. We present the theory of energy harvesting from the human body and describe the amount of energy that can be harvested from body heat and from motions of various parts of the body during walking, such as heel strike; ankle, knee, hip, shoulder, and elbow joint motion; and center of mass vertical motion.

**Methods:**

We evaluated major motions performed during walking and identified the amount of work the body expends and the portion of recoverable energy. During walking, there are phases of the motion at the joints where muscles act as brakes and energy is lost to the surroundings. During those phases of motion, the required braking force or torque can be replaced by an electrical generator, allowing energy to be harvested at the cost of only minimal additional effort. The amount of energy that can be harvested was estimated experimentally and from literature data. Recommendations for future directions are made on the basis of our results in combination with a review of state-of-the-art biomechanical energy harvesting devices and energy conversion methods.

**Results:**

For a device that uses center of mass motion, the maximum amount of energy that can be harvested is approximately 1 W per kilogram of device weight. For a person weighing 80 kg and walking at approximately 4 km/h, the power generation from the heel strike is approximately 2 W. For a joint-mounted device based on generative braking, the joints generating the most power are the knees (34 W) and the ankles (20 W).

**Conclusions:**

Our theoretical calculations align well with current device performance data. Our results suggest that the most energy can be harvested from the lower limb joints, but to do so efficiently, an innovative and light-weight mechanical design is needed. We also compared the option of carrying batteries to the metabolic cost of harvesting the energy, and examined the advantages of methods for conversion of mechanical energy into electrical energy.

## Background

### Motivation

With the increasing use of portable electronics, such as mobile phones, global positioning systems (GPS), and laptop computers, in our daily lives, the need for mobile electrical power sources is increasing. The power demand for the operation of these devices is typically met by batteries. However, the need to recharge batteries (or eventually to replace them) constitutes a significant limitation on the operating time (or lifespan) of portable electronic devices. For general use in the Western world, this problem is merely an inconvenience that can be solved by simply connecting the relevant device to an electrical grid. However, for some users, such as for those living in Third World countries or travelling in remote areas, this solution is not practical, as the power grid may not be well developed or stable.

The availability of efficient mobile electrical power sources would also be of significant benefit to users of computerized prostheses, such as the PROPIO FOOT^®^, RHEO KNEE^®^, and C-Leg^®^, which have an average power consumption of less than 1 W, but require charging at least every two days [1-3]. An even more power-demanding application is the Power Knee™, a powered prosthesis with actuation for above-knee amputees. Power Knee requires charging after every six hours of continuous use [4].

The convenience of all above applications would be enhanced by a technology that would provide energy for an extended time, without the need to recharge batteries. To date, developments to optimize power usage and produce batteries with better power density have resulted in an approximately twofold improvement in power density every decade [5]. Nevertheless, the operational usage time of any "off-the-electrical-grid" mobile system is limited by the requirements to carry and to recharge batteries. This drawback signals the need for further research on portable electrical generating devices that can increase both the amount and the usage time of electrical power.

A promising clean alternative way of meeting the above-described need is to exploit the heat and motions generated by the human body to generate electrical energy, and it is such a method that is investigated and reported in this paper. The objective of this paper is thus to present new insight into the theory of energy harvesting from the human body and to quantify the potential power of this source. Further, this paper reviews the currently available energy-harvesting devices, develops design guidelines, and provides recommendations for improving these designs.

The paper is structured as follows. The next section explains the theory and the logic underlying energy harvesting from humans by exploiting body heat and motions. Next, the Methods section shows how to estimate the magnitude of the potential energy in body movements both experimentally and from published data. The Results section provides estimations of the energy of such motions. The Discussion reviews device design considerations and the state of the art in energy conversion devices. Last, in the Conclusions section, limitations, challenges and future directions for technology development are discussed.

### The body as a source of energy - theoretical considerations

The idea of harvesting energy from human motion is based on the fact that an average person's energy expenditure, which is the amount of energy used by the body, is 1.07*10^7 ^J per day [6], an amount equivalent to approximately 800 AA (2500 mAh) batteries, whose total weight is about 20 kg. This energy is generated from energy dense sources. In comparison to batteries, this amount of energy can be produced from 0.2 kg of body fat. We note here that human energy is derived from food (carbohydrates, fats, and proteins), and the specific energy of food is typically 35 to 100 times more than the specific energy of currently available batteries (depending on the type of batteries used) [7].

The considerable amounts of human energy released from the body in the forms of heat and motion open the way for the development of technologies that can harvest this energy for powering electronic devices. The main challenge in developing such a technology lies in constructing a device that will harvest as much energy as possible while interfering only minimally with the natural functions of the body. Furthermore, such a device should ideally not increase the metabolic cost, i.e., the amount of energy required by a person to perform his/her activities.

**Figure 1:**
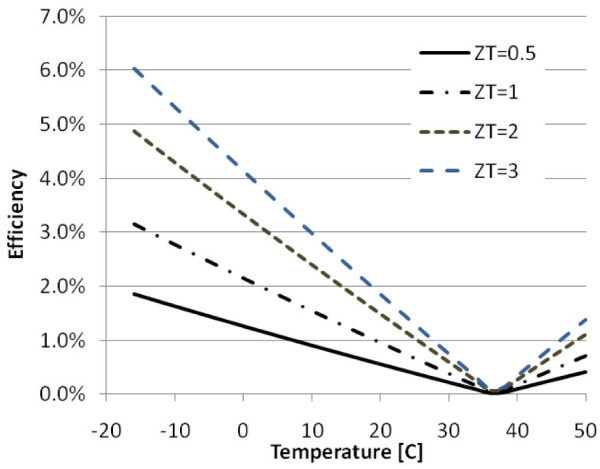
**Thermoelectric device efficiency as a function of the environment temperature and the figure of merit**. (body temperature assumed to be 37°C)

The mechanical efficiency of the human body is estimated to be about 15-30% [8], which means that most of the energy consumed as food is released into the atmosphere as heat. It therefore seems logical to attempt to harvest this thermal energy and convert it into electrical energy. Based on Carnot's equation [9], it is possible to calculate the maximum efficiency of a heat engine, which is a device that converts heat energy into mechanical energy. At an environmental temperature of 0°C, the optimal efficiency of such a heat-harvesting system would be:(1)

where *T*_*Body *_and *T*_*Ambient *_are the body and the surrounding temperatures in degrees Kelvin, respectively. The main technology for converting heat into electricity in this range of temperature differences is based on thermoelectric materials. The efficiency of thermoelectric devices is inferior to that of heat engines (as given by Carnot's equation) and is given by the following equation:(2)

where μ is the device efficiency, *T*_*h *_is the hot temperature, *T*_*c *_is the cold temperature, *ΔT = T*_*h*_*-T*_*c *_is the temperature difference, and ZT is the figure of merit for the device [10]. Among thermoelectric materials, alloys based on bismuth in combination with antimony, tellurium, or selenium are most suitable for use in devices for converting human body heat into electricity [11]. Typically, the figure of merit for thermoelectric generators is at best ZT≈1. Although only very slight improvements have been made to this figure of merit in the past few decades [12], the expected progress in the development of new materials with higher figures of merit could increase the efficiency of thermoelectric generators. Furthermore, it should be remembered that the efficiency of such devices depends on the temperature difference between the body and the surroundings, and therefore the greater the difference, the greater the increase in the efficiency and vice versa (Figure 1).

For an environmental temperature of 0°C and ZT = 1, equation (2) reveals that the actual efficiency of such a device would be approximately 2.15% (4% for a material with ZT = 3). Another important consideration in harvesting energy from the human body is the mechanisms through which heat is lost to the surroundings. The two modes of heat emission are heat transfer (sensible heat) and heat loss through evaporation (sweat = latent heat) (Table 1), but thermoelectric devices can exploit only the temperature difference, i.e., the sensible heat, and therefore latent heat, is "wasted."

**Table 1:** Human heat emission in different activities

	total (W)	sensible (W)	latent (W)
Seated at rest	100	60	40
Seated light work (writing)	120	65	55
Seated eating	170	75	95
Walking at 3 mph	305	100	205
Heavy work (lifting)	465	165	300
Athletics	525	185	340

Source: 1977 fundamentals, ASHRE Handbook & Product Directory ambient temp = 25.5°C

The total sensible heat that is released into the atmosphere by a person walking at natural speed is approximately 100 W [13]. If we could capture all this energy and convert it into electricity with an efficiency of 2.15%, the maximum power available during walking would be approximately 2 W. However, to harvest this energy, it would be necessary to cover the body with a thermoelectric material (perhaps a jacket or a garment like a diving suit). The design of an item of clothing with an embedded thermoelectric material that would cover part of the body (or the whole body) is obviously a challenge. Since in cold weather, the device would have to function as thermal insulator; however, currently available thermoelectric materials have a much higher thermal conductivity than typical coat material. This would result in a coat that would be too heavy to wear or in a need for an additional layer of thermal insulation material, thereby reducing the temperature difference along the device. In addition, such a device would have to allow sweat evaporation; however, this would mean that some of the sensible heat would flow out through the openings, causing a loss of available energy. The above data suggest that this technology would be more practical for low power applications, for which it would be necessary to cover only a small part of the body. One such example is the Seiko Thermic watch, which uses a thermoelectric material to generate its own power [14].

The relatively low power output of thermoelectric technology led us to consider the exploitation of the mechanical energy that can be derived from the body during motion to produce electrical energy. When considering a particular motion as a candidate for energy harvesting, the following main factors must be taken into consideration. First, muscles perform positive and negative mechanical work within each motion: During the positive work phase, the muscles generate the motion, and in negative work phases, the muscles absorb energy and act as brakes to retard or stop the motion. Winter [8] defined negative and positive muscle work as follows: Positive work is the work performed by the muscles during a concentric contraction, i.e., shortening of the muscle when the torque applied by the muscle at the joint acts in the same direction as the angular velocity of the joint. When the muscle performs positive work, it generates motion. Therefore, the use of positive energy (e.g., turning a crank to generate electricity) is will always increase the metabolic cost. On the other hand, negative work is the work done during an eccentric contraction, i.e., lengthening of the muscle, when the muscle torque acts in the direction opposite to the angular velocity of the joint. An energy harvesting device should therefore replace part of the muscle action during negative work and create resistance to retard the motion, similar to "generative braking" in hybrid cars. Theoretically, such a device will allow energy generation with minimal or no interference with natural motions.

In this paper, we explore the option of generating energy during activities that are performed naturally throughout the day, with particular emphasis on walking. The choice of walking as a candidate movement for the study of energy harvesting is based on the fact that it is a natural movement, performed without conscious thought and involving a range of relative motions between different body segments and between different segments and the ground. When assessing the potential power harvesting capability of an energy-harvesting device, we must consider five main factors: the muscle's negative work phases during each motion, the means by which the device is attached to the body, the convenience of use of the device, the effect of the additional weight of the device on the amount of effort expended by the wearer, and finally the effect of the harvesting energy device on the body. For example, during walking, in the heel strike phase, energy is converted into heat in the shoe sole [15], and harvesting this energy should not affect the normal gait pattern.

In the following section, we will analyze the main body motion segments during natural walking to facilitate assessment of the potential power-harvesting capability during each motion segment.

## Methods

The major body motions during walking that we considered as potential energy sources were heel strikes, center of mass motion, shoulder and elbow joint motion during arm swings, and leg motions, i.e., ankle, knee, and hip motions. To estimate the potential power of each motion, we performed an integrative analysis using data available in the literature. In addition, for the upper body joints we conducted our own experiment to calculate the power of each motion.

For the analysis of the energy produced during the above-described motions, we used two definitions of work: 1) the force acting through a displacement, and 2) the product of torque and angular displacement.(3)(4)

where force and torque are denoted as F and τ, respectively, and the linear and angular displacements are denoted as S and θ, respectively.

Next, we analyzed each of these body motions and estimated the amount of work performed at the relevant joints/locations and the sign of the work (positive or negative) during walking.

### Heel strike

Heel strike refers to the part of the gait cycle during which the heel of the forward limb makes contact with the ground. Several researchers, e.g., [16], have modelled this motion as a perfect plastic collision, while others believe that there is an elastic component to this motion, e.g., [17,18]. It is, however, generally agreed that energy is lost during the collision. A number of researchers have tried to estimate the amount of energy dissipated in the collision. For example, Shorten [18] calculated the energy loss in a running shoe and related it to a force acting through a linear displacement. Using a viscoelastic model for the midsole, he determined the part of the energy is stored as elastic energy in the sole of the shoe and the part that is dissipated. He predicted that for a typical runner moving at 4.5 m/s, the value of the dissipated energy could range from 1.72 to 10.32 J during a single step and that most of the energy loss would occur during the heel strike.

To gain a better understanding of the source of energy, let us consider a simple model in which an external force acts on the sole of the shoe over a complete stride. The maximum ground reaction force acting on the shoe is approximately equal to 1.2 times the body weight, and most of the heel compression occurs directly after the heel strike (during the first 20% of the gait cycle). Therefore, assuming a displacement of 4 mm in the shoe sole and a body weight of 80 kg, we can calculate the work for the compression of the heel as approximately 2 J/step. Since a complete stride at natural walking speed has a frequency of approximately 1 Hz (two steps per second), the theoretical maximal power will be 4 W. Moreover, if 50-80% of the energy during walking is stored as elastic energy in the shoe [18], then the maximum energy that is available for use would be approximately 2W. While it is possible to construct a device that will have a larger displacement during the heel strike, such a design may impair stability and manoeuvrability [7]. Intuitively speaking, this will result in the wearer of the device feeling as if s(he) is walking on soft sand.

### Leg motion

**Figure 2:**
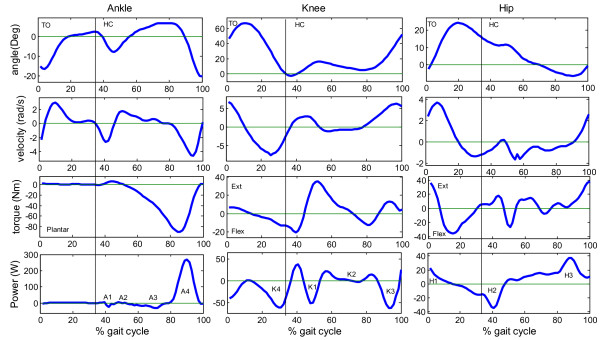
**Typical kinematics and kinetics during a walking cycle**. (subject mass = 58 kg, speed 1.3 m/s; cycle frequency 0.9 Hz. In data from [8]: zero ankle angle is defined as 90° between the shank and the foot; zero knee angle is full extension of the knee (straight leg); zero hip angle is with the thigh at 90° with the ground.

During walking, muscles generate torques at the ankle, knee, and hip joints. These torques acts along three axes (3-D), and their magnitude changes during the gait cycle (Figure 2). The most significant torques in terms of the work that is performed during the walking cycle are those acting in the axes normal to the sagittal plane [19]. Winter and colleagues [20] calculated the work performed at different leg joints during a single step and normalized it by the subject's weight. In addition, they divided the net work done by the muscles at the joints into several phases of motion. Their classification was based on the negative and positive muscle work performed at the joints during walking (Table 2). We used these findings to estimate the total work and the negative work performed during a gait cycle at the hip, knee, and ankle joints.

**Table 2:** Work performed at the leg joints during a walking step normalized by the subject's mass.

work during the phase (J/kg)	average (J/kg)	standard deviation (J/kg)
Ankle A-1	-0.0074	0.0072
Ankle A-2	0.0036	0.0046
Ankle A-3	-0.111	0.042
Ankle A-4	0.296	0.051
Knee K-1	-0.048	0.032
Knee K-2	0.0186	0.026
Knee K-3	-0.047	0.015
Knee K-4	-0.114	0.015
Hip H-1	0.103	0.047
Hip H-2	-0.044	0.029
Hip H-3	0.090	0.027

A1-4 are phases of work that are performed in the ankle joint, K1-4 are phases for the knee, and H1-3 are for the hip joint. Work represents the net summation of work at the joint muscles [20], and negative values represent negative work.

For an 80-kg person walking at normal speed, the joint work for each step is calculated by using the following equation:(5)

where the phases used for each joint calculation are based on the findings of Winter et al. [20], and the units are J/step.

#### Energy calculation for the ankle

Thus, the total energy is 33.4 J, and the negative portion is 9.7 J.

#### Energy calculation for the knee

Thus, the total energy is 18.2 J, and the negative portion is 16.7 J.

#### Energy calculation for the hip

Thus, the total energy is 18.96 J, while its negative portion is 3.52 J.

### Center of mass motion

Another motion that could be utilized to generate energy is the motion of the center of mass. The center of mass performs a motion similar to a 3-D wave (i.e., up-down and left-right). The total motion of the vertical wave from the lowest to the highest point is approximately 5 cm [8]. For an external mass (e.g., a backpack) to move with the body's center of mass, there must be work that is applied to this mass causing it to follow the human center of mass trajectory. To facilitate energy harvesting, there must be a relative motion between the mass and the person carrying it.

We used the following model to estimate an upper bound on the total amount of energy required to generate this motion, based on changes in the height of the mass in each gait cycle (i.e., for the mass moving up and down by approximately 5 cm during each cycle). Assuming no exchange of kinetic and potential energy, we used the following equation for the energy required to move the mass during one gait cycle: E = 2m·g·h, where E is energy, m is mass, g is gravitation acceleration, and h is height. By applying this equation for a center of mass motion of 5 cm during walking, we find that for a device of 20 kg there is a potential of 20 W to be harvested.

### Arm motion

Arm motion refers to the backward and forward swinging movement of the arm that occurs during walking and running. The arm motion is composed of two sub-motions: the relative motion between the forearm and the upper arm (change of angle of the elbow) and the relative motion between the trunk and the upper arm (change of angle at the shoulder).

To calculate the net muscle joint torque during the during the gait cycle, we used a recursive inverse dynamic (top down). Then, using the angular displacement and the joint torque (equation 3), we calculated the work at the shoulder and elbow joints during the gait cycle, according to the method applied by Winter and his colleagues for leg joints [20].

### Experiment to obtain data for arm energetics calculations

To calculate the energetics of the arm joints, we performed an experiment with three male subjects of average weight 82 kg (range 72-88 kg) and average height 1.80 m (range 1.72-1.86 m), who walked at a natural speed of 1.1 m/s (range 1.0-1.2 m/s). Motion data were obtained using a six-camera motion capture system at a sampling rate of 100 Hz (Vicon 460, Lake Forest, CA). Marker motion data were low-pass filtered (Butterworth fourth-order forward and backward passes) with a cut-off frequency of 6 Hz. The arm was represented by a two-link system, consisting of the upper arm and the forearm (including the hand). The segmental properties (mass, center of mass, and moment of inertia) were calculated on the basis of De Leva's adjustments [21] to the work of Zatiorsky-Seluyanov. The measurements from our experiment were used to calculate arm energetics.

## Results

A summary of our analyses is given in Table 3. This summary presents the amount of work performed in each joint or body part and of the portion that is negative work. Further, it shows the maximum joint torque during these motions; this information is required because for harvesting maximum energy, an energy conversion device should be able to withstand torques similar in magnitude to the maximum joint torque.

**Table 3:** Summary of total work done by the muscles at each joint or segment of the body during the walking cycle

joint	work [J]	power [W]	max torque [Nm]	negative work	
				%	J
Heel strike	1-5	2-20		50	1-10
Ankle	33.4	66.8	140	28.3	19
Knee	18.2	36.4	40	92	33.5
Hip	18.96	38	40-80	19	7.2
Center of mass	10**	20**		***	
Elbow	1.07	2.1	1-2	37	0.8
Shoulder	1.1	2.2	1-2	61	1.3

(*) Except for calculations for center of mass and heel strike, all other calculations were performed for an 80-kg person, assuming a walking frequency of 1 Hz per cycle (i.e., two steps). We chose to use 1 Hz to simplify the calculation, since it is close to the 0.925 Hz that was measured by Winter et al. [20].

** Energetic cost of transporting a 20-kg payload using two models (walking frequency of 1 Hz per cycle).

*** Center of mass also includes muscle negative work, but the magnitude is not known.

## Discussion

### Considerations for device design

We obtained results showing the amount of positive and negative muscle work in each motion, and motion where energy is lost to the surroundings (e.g., heel strike). The importance of these results is that they will affect the design of energy-harvesting devices.

It is possible to consider the harvesting of energy during positive work; for example, a user rotating a crank to generate energy. This type of generation of electrical energy would require an additional metabolic cost. Typically, muscle efficiencies during positive work are approximately 25%, which means that if all the mechanical work were converted into electricity, there would be an increase of approximately 4 J of metabolic cost for every 1 J of energy generated. A better way to generate energy from human motion would be to use energy that would otherwise be lost to the surroundings. This would ideally enable the generation of electricity from human motion with minimal or no additional load. There are two types of motion relevant to energy harvesting: 1) motion in which energy is lost directly to the surroundings (e.g., heel strike) in the form of heat, plastic deformation, sound, or other forms, and 2) motion in which the muscles perform negative work. Exploiting the latter type of motion in an energy-conversion device might not cause an additional load to the user. The idea explored in this paper is that in these phases the muscles act as brakes to slow down the motion of the limb. By replacing the negative work done by the muscle with an electric generator, we can reduce the load on the muscles and generate electricity at the same time.

Another important consideration is the way in which this motion is utilized. For example, while the knee and elbow joint motions are mostly single-degree-of-freedom movements, the shoulder and the hip joints perform much more complex movements, and, therefore, much more complex mechanisms would be required to exploit the energy generated from these joints. Consequently, we focus on joints with one-degree-of-freedom motion. In addition, it is important to know the maximum joint torque during these motions, since for maximum energy harvesting, an energy-conversion device should be able to withstand torques of similar magnitude to maximum joint torque. A torque of higher magnitude on the device would require stronger transmission and would therefore lead to an increase in the device weight, which would, in turn, increase the energy expenditure. Moreover, the lower the additional mass mounted on the leg, the higher the energetic cost of carrying it [22,23].

From our analysis of human motions during walking (Table 3), we can see that all the motions examined include some negative-energy phase. For an energy-hungry application, we need to maximize the total amount of energy to be harvested, and, therefore, heel strike, and knee and ankle motions seem to be good candidates for energy harvesting devices, since a relatively large part of their total energy can be recovered. Furthermore, these motions are almost all single-degree-of-freedom movements, which simplifies the device design.

#### Efficiency of harvesting electrical power

The magnitude of the power that can be harvested is not the sole consideration for choosing a movement or designing a device; the other important parameter for an energy-harvesting device is its efficiency.(6)

where *Δelectrical_power *is the electrical power output and *Δmetabolic_power *is the difference in metabolic cost of a particular activity with and without a device (e.g., walking with a device and without it). The change in metabolic cost is made up of two main components: 1) the energy spent to generate the electrical power, and 2) the energy spent by the user in carrying the device, which is a function of the device weight and the location of the device on the body. Therefore, in a comparison of two devices, the efficiency of harvesting might be a better metric than the maximum power output. For example, for two devices of equal weight producing the same amount of energy, a knee device will have better efficiency than an ankle device because the cost of carrying the knee device mass is lower. Note that a reduction of the device weight by the use of lighter materials (e.g., carbon fibers) and an optimized design will also reduce the cost of carrying the device and will lead to the development of more efficient devices. The first component of the change in human metabolic power derives from the generation of electrical power. This addition in metabolic power is affected by muscle work and device conversion efficacy [24].(7)

Where Δ*Metabolic power*_*g *_is the change in metabolic power due to the change in muscle work resulting from the energy generation component alone, *η*_*device *_is the device efficiency, and *η*_*muscle *_is the muscle efficiency in the given motion.

The change in metabolic cost due to the change in muscle work is dependent on the type of work done by the muscles, since the efficiencies of positive and negative work at the joint are not the same. For positive work, the efficiency ranges between 15% and 25% [8], while for negative work, the values range from 28% to 160% [25,26]. The parameters that affect muscle efficiencies are: the nature of the performed motion, the particular muscles involved, and the activation forces and velocity of these muscles. This means that when the energy harvester replaces the muscle work during negative work, the predicted reduction in metabolic cost will be less than the predicted reduction for replacing positive work phases. In addition, in some cases, the negative work is performed using passive elements such as connective tissue, which store elastic energy like springs and return it back to the gait cycle [27]. In these cases, harvesting this energy might mean that the muscles will have to perform extra work in order to replace the energy that is lost to the device. For devices based on generative braking, we used the joint net power as a criterion to determine which joints are good candidates for energy-harvesting devices. It is, however, difficult to interpret the contribution of each muscle to the net joint torques, for the following reasons: 1) muscles work across multiple joints, and therefore, theoretically, it is possible that a particular muscle will contribute to negative work at one joint and positive work at another; and 2) the net joint torque is a function of all the activity of agonist and antagonist muscles and as such cannot account for simultaneous generation of energy by a certain muscle group and absorption by the antagonist group, or vice versa. As a result, it is possible that when the generator resists motion during positive power, it will help the muscle that is doing negative work. Therefore, recommendations as to the appropriate joint to be exploited for generative braking based on the amount of negative work done at the joint should be considered only as guidelines, and the final evaluation must be based on experimental work.

#### Comparing the cost of energy harvesting to carrying batteries

While ideally the energy-harvesting device should not increase the metabolic cost, it is possible that in some cases it will do so. In these cases, the user may have to consume extra food to cover the additional metabolic cost for electricity generation. Hence, for a given mission, the best option should be chosen on the basis of a comparison between the metabolic cost for generating energy and carrying extra food versus carrying batteries with the equivalent amount of energy. In the case of a backpack device [7], the user carries the food and batteries on his/her back, and thus the cost of carrying the weight is the same for both. In this regard, Rome et al. [7] reported a device that achieved 19.5% efficiency in converting metabolic energy to electrical power. Since the specific energy of food is typically 3.9 × 10^7 ^J kg^-1 ^[28], which is much greater than the specific energy for lithium batteries (4.1 × 10^5 ^J kg^-1^) and zinc-air batteries (1.1 × 10^6 ^J kg^-1^) [29], the weight of food would be 19 times lighter than that of lithium batteries and 7 times lighter than that of zinc-air batteries. Therefore, they concluded that the addition of food weight is negligible. This means, for example, that walking at 1.5 m/s (while generating 5 W) for 10 h would save approximately 0.4 kg of lithium batteries and 0.15 kg of zinc-air batteries, meaning the longer the expedition, the greater the weight savings.

Now that we have estimated the potential of energy to be harvested from each of the body motions and discussed considerations in utilizing energy sources from human motion, we believe it is important to include a review of existing devices. These devices are classified according to the motions used to harvest the energy and their location on the body.

### Review of the state of the art in energy-harvesting devices

#### Center of mass

**Figure 3:**
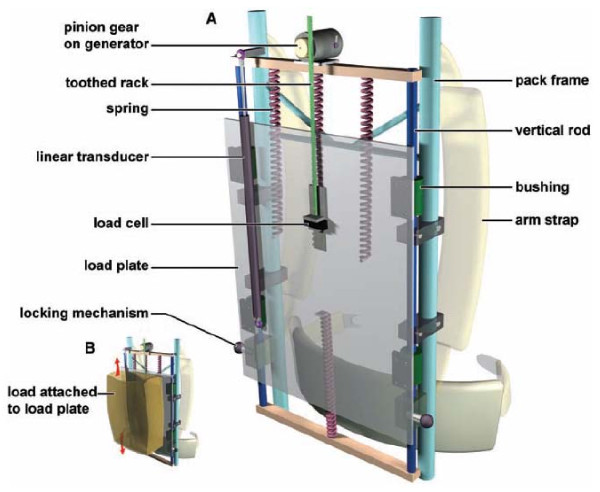
**Suspended-load backpack for generating energy**. The pack frame is fixed to the body, but the load is mounted on a load plate and is suspended by springs (red) from the frame (blue) (A). During walking, the load is free to ride up and down on bushings constrained to vertical rods (B). Electricity generation is accomplished by attaching a toothed rack to the load plate, which (when moving up and down during walking) meshes with a pinion gear mounted on a geared dc motor, functioning as a generator. The motor is rigidly attached to the backpack frame [12]. (Reprinted with permission from Science Incorporated.)

Currently available center-of-mass devices use the motion of the center of mass relative to the ground during walking to generate energy. For example, when carrying a backpack, the body applies forces on the backpack or any other mass in order to change the direction of its motion. Rome and colleagues used these forces in a spring-loaded backpack that harnesses vertical oscillations to harvest energy [7]. This device, with a 38 kg load, generates as much as 7.4 W during fast walking (approximately 6.5 km/h). The device is a suspended-load backpack (Figure 3) that is interposed between the body and the load, resulting in relative motion movement. For this device, the relative motion was approximately 5 cm, and this linear motion was converted into rotary motion that drove a generator (a 25:1 geared motor). Generation of this energy was achieved with the small amount of extra metabolic cost of 19 W, which is 3.2% more than carrying a load in regular backpack mode (with no relative motion). This additional cost is less than 40% of that required by conventional human power generation (e.g., hand-crank generators or wind-up flashlights). While the mechanism of this energy harvesting is not fully understood, from the above results it seems reasonable to believe that there is contribution of both negative and positive muscle work.

Another approach to harvesting energy using a backpack was taken by Granstrom and colleagues [30], who mounted a piezoelectric material in the shoulder strap of a 44-kg backpack and used the stress in the straps to generate 50 mW. A different class of device that uses the motion of the center of mass to harness energy is based on oscillations of a floating magnet due to this motion. Niu and colleagues built a linear electrical generator (1 kg) that used the motion of the body during walking to produce 90-780 mW, depending on the walking conditions [31]. They optimized the electrical circuits and linear generator design to produce the highest power output from the walking motion.

### Heel strike

Several devices have been built to generate energy from heel-strike motion. Some devices use the energy from the relative motion between the foot and the ground during the stance phase (the phase in which the foot is on the ground). Others use the energy from the bending of the shoe sole. In both cases, the device aims to use the energy that would otherwise have been lost to the surroundings. An example of such a device is a hydraulic reservoir with an integrated electrical magnetic generator that uses the difference in pressure distribution on the shoe sole to generate a flow during the gait cycle. This prototype produces an average power of 250-700 mW during walking (depending on the user's gait and weight); its drawback is that it is quite bulky and heavy [32]. Paradiso and his colleagues [33] built a shoe that harvests energy using piezo-electric materials from heel strike and the toe off motions. The average power during a gait cycle is 8.3 mW. Another device that was built by the same group is a shoe with a magnetic rotary device that produces a maximum power of 1.61 W during the heel strike and an average power of 58.1 mW across the entire gait.

A different approach was taken by Kornbluh and his collaborators [34] at SRI International, who developed electrostatic generators based on electroactive polymers (EAPs). Such materials can generate electricity as a function of mechanical strain. Their technology provides energy densities for practical devices of 0.2 J/g. In addition, these materials can "cope" with relatively large strains (50-100%). The SRI team incorporated an elastomer generator into a boot heel. Their generator design was based on a membrane that is inflated by the heel strike. They achieved 0.8 J/step (800 mW) with this device. The energy was harvested during a compression of 3 mm of the heel of the boot onto which the device was mounted [34]. A key advantage in the construction of such devices is that they can be mounted on an existing shoe, thereby obviating the need for a special external device to generate energy. The power output of these devices is relatively low, with a maximum of approximately 2 W at normal walking speed. However, there are many applications (e.g., MP3 players, PDA, cellular telephones) for which this energy would be sufficient to operate the device.

### Knee

**Figure 4:**
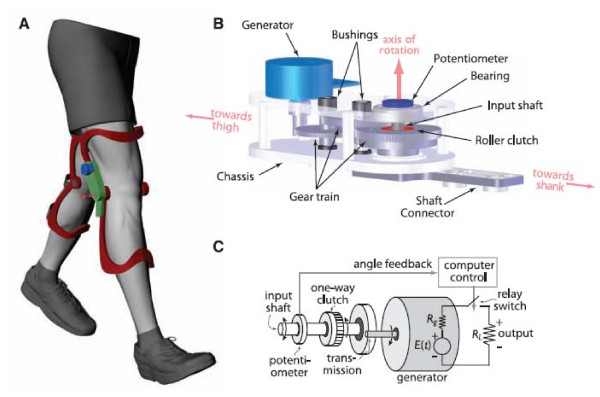
**Biomechanical knee energy harvester **[24]. (A) The device has an aluminium chassis and generator (blue) mounted on a customized orthopedic knee brace, totalling 1.6 kg; one such brace is worn on each leg. (B) The chassis contains a gear train that converts the low velocity and high torque of the knee motion into the high velocity and low torque required for the generator operation, with a one-way clutch that allows for selective engagement of the gear train only during knee extension and no engagement during knee flexion. (C) The schematic diagram shows how a computer-controlled feedback system determines when to generate power using knee-angle feedback, measured with a potentiometer mounted on the input shaft. Generated power is dissipated in resistors. *Rg*, generator internal resistance; *R*_*L*_, output load resistance; E(t), generated voltage. (Reprinted with permission from Science Incorporated.)

A device for the knee joint based on negative work of the muscles was proposed by Niu and colleagues [35] and subsequently developed by Donelan et al. [24,36]. This 1.6-kg device comprised an orthopedic knee brace configured such that knee motion drove a gear train (113:1) through a unidirectional clutch, transmitting only knee extension motion to a DC brushless motor that served as the generator (Figure 4). The generated electrical power was dissipated by a load resistor. This method generated 2.5 W per knee at a walking speed of 1.5 m/s. The additional metabolic cost of generating energy (not including the cost of carrying the device) was 4.8 W, i.e., 12.5% of the metabolic cost required by conventional human power generation. However, there were certain drawbacks associated with this device in that it used only a small part of the motion of the knee (end of the swing phase) to generate energy: During the gait cycle, the muscle net work in the knee joint is approximately 90% negative work, which is approximately 34 W, but the device harvested energy only at the end of swing phase and with an efficiency of 65%. Based on this data, we calculated the difference between the power of the current device and that of an ideal device (that would harvest all the negative work during walking). The power that is still available = (total power - current power output/efficiency)*device efficiency = (33.5-5/0.65) × 0.65 = 16.8 W. The main challenge in harvesting energy from the knee movement is that as more energy is harvested, the resistance to the motion as generated by the device will increase, thereby increasing the motion controls by the device at the expense of the muscles.

### Method for energy conversion

A key component of the energy-harvesting devices reviewed above is the method they use to convert the mechanical work to electricity. The main technologies in current use are based on piezoelectrics, EAPs, and electrical induction generators. Piezoelectric materials, which generate a voltage when compressed or bent [38], have been used mainly for heel strike devices. Their main advantage is that they are simple to incorporate into a shoe. However, due to the small displacement and the high generated voltage, the power output of this technology is limited to approximately 100 mW [35]. EAPs also generate electricity when under mechanical stress, but they have a low efficiency (compared to magnetic machines) and a relatively high operation voltage, both of which can make the electrical circuit complicated and expensive. Yet, due to the excellent strain properties of EAPs when compared to piezoelectrics, more energy can be harvested from the former. Furthermore, EAPs are much lighter and easier to shape than magnetic materials. Therefore, we conclude that EAPs are a good alternative to piezoelectrics for biomechanical applications [38]. Of the three technologies discussed above, magnetic machines, which are low in cost, have the highest conversation efficiency. However, the higher efficiency levels are generally achieved at high speeds and in rotary implementations. Human motions, in contrast, are relatively slow, and, as a result, the application of electromagnetic energy conversion needs an addition of transmission to increase the rotation speed. While for the backpack, the transmission adds only a small percentage to the total weight, in the knee device, the transmission construction added approximately 650 g, which was 40% of the total weight of the device. Moreover, when using a rotary magnetic-based generator, the input should ideally have a constant rotation direction and speed. However, human joint angles change speed and direction during the walking cycle, which adds complexity to the use of rotary magnetic devices to harvest energy.

Possible directions for future research are the innovative design of magnetic machines that reduce the need for high rotary speeds, improvement of the power density of elastomers and magnetic-based generators (e.g., using stronger magnets), and improvement of the efficiency of energy harvesting by using elastomers.

## Conclusions

Biomechanical energy harvesting technology is an innovative approach for producing energy for portable devices. Here, we have used biomechanical models to estimate the potential power output that could be harvested from each of the major human motions and have discussed the advantages and disadvantages of exploiting each motion. Further, a review of the state of the art in this technology and types of energy conversion methods reveal that for heel-strike devices the most promising technology seems to lie with EAPs, which have a high power-to-weight ratio and produce energy in the amount of 0.8 W, i.e., close to our estimation of a maximum of 2 W during normal walking. The utilization of center of mass motion enables the production of energy with 40% of metabolic cost of generating the energy using conventional energy harvesting, such as wind-up flashlights that use positive muscle work. Devices of this type utilize energy from the relative motion between a mass and the human body to generate mechanical power, which is then converted into electrical power. Therefore, the amount of energy that can be produced depends on the weight of the moving mass.

The newest technology for energy harvesting is generative braking (similar to that used in hybrid cars), thereby replacing muscle work. Theoretically, this method has potential to generate 60 W when considering all the leg joints; however, typical conversion losses are 50%, and therefore it is reasonable to believe that it is possible to generate 25 W at a normal walking pace (approximately 4 km/h). This is a greater amount of available energy than the energy that could be produced by other methods without increasing the metabolic cost. However, generative braking is not easy to implement: the main challenges that must be overcome to reach this goal are discussed below.

First, the currently available knee device works only at the swing phase; thus, all the phases of negative work during the gait cycle are not utilized. The main challenges in harvesting all the phases lie in the changes in the speed and direction of the joint angles throughout the walking cycle, during which the generator should ideally rotate at constant speed and direction. To achieve a constant direction of rotation, there is a need for a light mechanism that would accept rotation input in both directions and yield an output rotation in one direction. In addition, a gear with a changing transmission ratio to keep the generator rotation speed constant would be required.

Second, a high gear ratio is required. A rotary magnetic-based generator typically rotates at a high speed (1000-10,000 rpm), while the human angular velocity for a typical joint is of the order of 20 rpm. Yet, the higher the gear ratio, the greater the losses due to friction, and the higher the weight of the device. These considerations, together with the metabolic cost of carrying additional weight, call for an innovative and lighter design.

Third, another area that must be developed is the area of control. Current devices use an on/off control with a load that, for a given motion, is determined by the generator, the gear ratio, and the effective electrical load. To improve the amount of energy that can be harvested, there is a need to match the angular and torque curves of the generator to replace the torques that are normally produced by the joint muscles during a given motion. There are two ways to do this: first, by constantly changing the gear ratio, and second, by changing the effective external electrical load. However, a high power output means that the greater part of the motion control falls on the device rather than on the muscles, and this would require a much more sophisticated control mechanism. Currently,, the knee harvester was tested during walking on a flat surface (treadmill), and the angular velocity data was used to control the timing of harvesting. Yet, for walking on a terrain that alters the gait pattern, angular data might not be sufficient to determine the joint negative power phase.

In summary, biomechanical energy harvesting constitutes a clean, portable energy alternative to conventional batteries for electronic mobile devices. This is especially true for areas where the power grid is not well developed, such as in Third World countries. In addition, this technology could serve as a power source for devices with low power requirements. High-power medical devices, such as prostheses with electrical motors and controllers and exoskeletons, could certainly also benefit from the development of this technology.

## Abbreviations

Cm: centimetre, a length measurement unit; EAP: Electro Active Polymers; g: The acceleration of gravity.; kg: Kilogram; GPS: Global Positioning system; Hz: Hertz, a frequency measurement unit; J: Joule, an energy measurement unit; km: Kilometre, a distance measurement unit; m: Meter, a length measurement unit; MP3: Audio payer, base on compression technology of 3 Layer from Moving Picture Experts Group.; PDA: Personal Digital Assistant; W: Watt, a power measurement unit

## Competing interests

The authors declare that they have no competing interests.

## Authors' contributions

RR took the lead role in the biomechanical analysis and the human physiology, mechanical and figure design, and manuscript writing. AS contributed to the aspects of mechanical design and control that are related to this study and to the writing. Both authors have read and approved the final manuscript.
